# Extending the Branches of Neonatal Neurocritical Care

**DOI:** 10.1016/j.semperi.2025.152131

**Published:** 2025-09-18

**Authors:** Mark A. Petersen, Hannah C. Glass

**Affiliations:** a Department of Pediatrics, University of California San Francisco, San Francisco, CA, USA; b Department of Neurology and Weill Institute for Neuroscience, and Department of Epidemiology and Biostatistics, University of California San Francisco, San Francisco, CA, USA

**Keywords:** Neuromonitoring, Neuroimaging, Neurodevelopmental outcomes, Artificial intelligence/machine learning, Neuroprotection, Stem cells, Neurorepair

## Abstract

Neonatal Neurocritical Care (NNCC) is transforming neonatal medicine through a brain-centered approach that reflects the complexity and rapid progression of early neurodevelopment. NNCC provides a comprehensive care model built on a foundation of real-time neuromonitoring, advanced neuroimaging, and multidisciplinary collaboration to deliver timely neurological support for newborns at risk of injury. This framework has evolved to bridge the full arc of early brain development, linking fetal evaluation, neonatal management, and post-discharge follow-up into a cohesive continuum. Recent technological advances in bedside monitoring, functional neuroimaging, machine learning, and rapid genomics are shifting NNCC toward a more proactive and personalized model of care. Emerging therapies are also expanding the clinical reach of NNCC, opening new frontiers for early intervention and neurorepair. As the field continues to advance, the central goal of NNCC remains to improve neurodevelopmental outcomes while ensuring widespread access to high-quality, family-centered neurological care. This review outlines the evolving landscape of NNCC and highlights its essential role in guiding brain-focused care from the earliest stages of development through childhood and beyond.

## Introduction

Neonatal Neurocritical Care (NNCC) has emerged as a transformative framework for supporting brain health in critically ill newborns. NNCC was developed to improve neurological outcomes in high-risk neonates through early identification of brain injury, expanded use of neuromonitoring and imaging, and, importantly, timely interventions at the bedside such as early seizure management and therapeutic hypothermia. Central to NNCC is the close collaboration between neonatologists, pediatric neurologists, and specially trained neonatal nurses, who work together to interpret real-time monitoring, imaging, and clinical assessments and deliver personalized neuroprotective care.^1–6^ Over time, NNCC has evolved into a fully integrated, multidisciplinary field that spans neonatology, neurology, radiology, neurophysiology, neurosurgery, and developmental care. This shift reflects a growing understanding that brain-centered care, when applied early and consistently, can shape not only short-term outcomes but also long-term neurological and cognitive trajectories.

Today, NNCC is expanding beyond a model focused solely on mitigating injury to also encompass prevention, precision, and repair. Advances in fetal neuroimaging and maternal-fetal therapies are pushing the boundaries of care earlier into the prenatal period. At the same time, real-time multimodal bedside monitoring, enhanced by artificial intelligence, is beginning to reshape how clinicians assess and respond to neurological risk in the newborn. Emerging therapeutic strategies, including anti-oxidant and anti-inflammatory medications for neuroprotection, blood-targeted interventions for intraventricular hemorrhage (IVH), myelin-promoting agents for white matter injury (WMI), and stem cell therapies for neonatal stroke and hypoxic-ischemic encephalopathy (HIE), are creating new opportunities for early intervention and neurorepair. NNCC now also extends beyond the acute care setting to encompass long-term neurodevelopmental care. Early brain injury is strongly associated with later challenges in cognition, motor function, behavior, and school readiness.^7–11^ As such, NNCC is increasingly connected to early intervention services, developmental monitoring, and strategies that link neonatal brain health with long-term potential. This expanding scope calls for an integrated continuum of care that begins in the prenatal period and extends through infancy and beyond, ensuring that all newborns have access to high-quality neurological care regardless of where they are born.

**Fig. 1.**
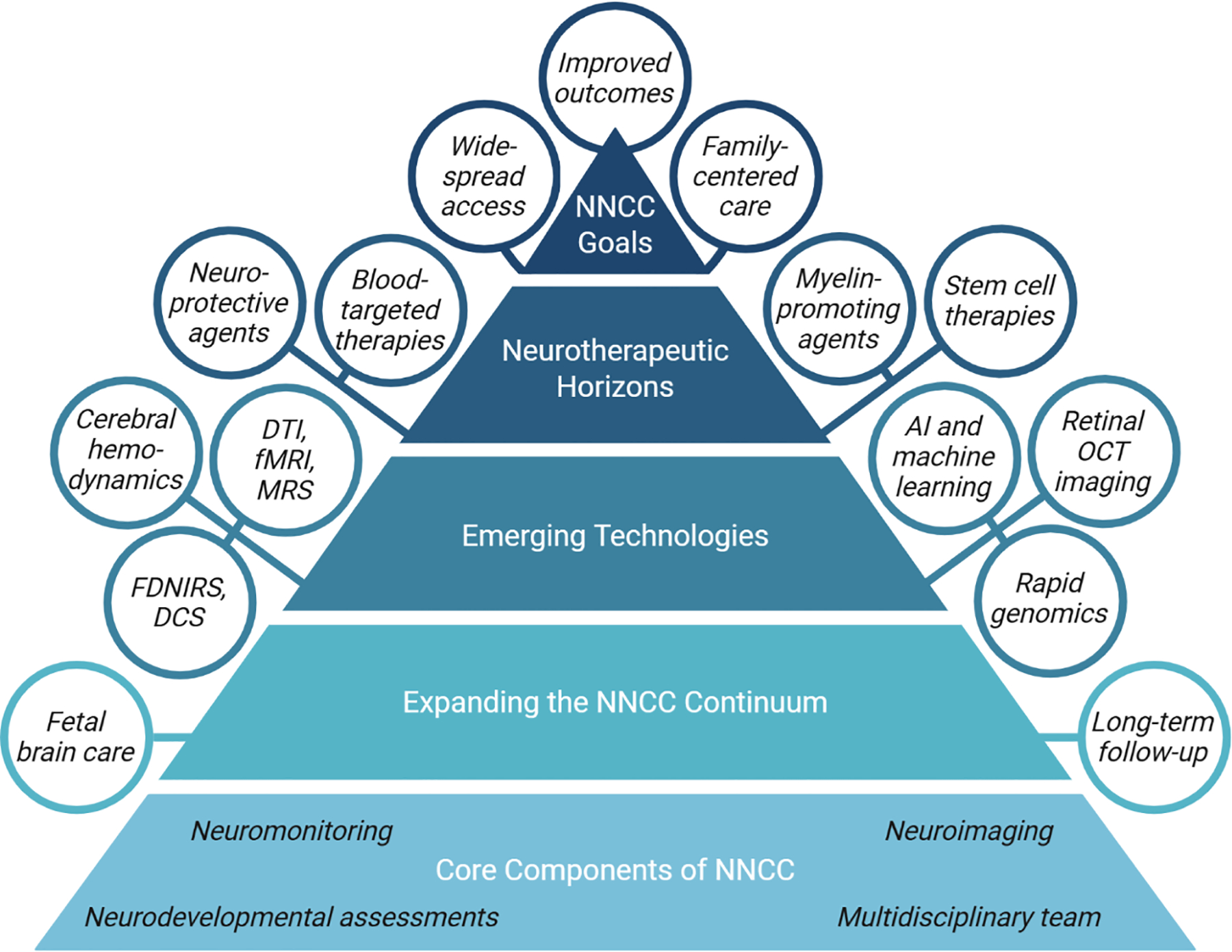
Extending the branches of neonatal neurocritical care (NNCC). AI: artificial intelligence, DCS: diffuse correlation spectroscopy, DTI: diffusion tensor imaging, FDNIRS: frequency-domain near-infrared spectroscopy, fMRI: functional magnetic resonance imaging, MRS: magnetic resonance spectroscopy, OCT: optical coherence tomography. Created in BioRender. Petersen, M. (2025) https://BioRender.com/8so0lds.

This review explores the expanding landscape of NNCC, building on insights gained since the development of the first dedicated Neuro-Intensive Care Nurseries (NICNs) / Neuro-Neonatal Intensive Care Units (Neuro-NICUs). It traces how the field has evolved, translating foundational advances in continuous neuromonitoring, longitudinal neuroimaging, and human neuropathology into a multidisciplinary framework that now integrates fetal brain assessment, new technologies, emerging neurotherapies, and long-term developmental follow-up (Fig. 1). NNCC has established itself as an essential component of neonatal medicine, advancing neuro-focused and family-centered interdisciplinary care to improve both short- and long-term outcomes.

## Core components of NNCC

The first NNCC units were developed to address critical gaps in the neurological care of high-risk newborns (Box 1). The model arose from a growing recognition that early brain injuries were often undetected or inconsistently managed in traditional neonatal intensive care units (NICUs). In response, a structured, evidence-based framework was created to proactively and collaboratively manage neurological conditions in neonates.^1–6^ From this foundation emerged a set of core clinical tools and approaches, including continuous neuromonitoring, neuroimaging, and longitudinal neurological assessments, that now guide nearly every brain-focused decision in neonatal care. Grounded in multidisciplinary collaboration and supported by clinical guidelines, the NNCC model enables timely intervention, promotes consistency, and delivers comprehensive neuroprotective care through seizure management, optimization of cerebral oxygenation and perfusion, targeted temperature management, correction of glucose and electrolyte disturbances, and coordination with developmental support services.^1–6^

### Neuromonitoring

Neuromonitoring at the bedside provides real-time insight into neonatal brain function. Continuous electroencephalogram (cEEG) and amplitude-integrated EEG (aEEG) are widely used to detect and manage seizures in conditions such as HIE, stroke, intracranial infection, metabolic encephalopathies, and prematurity-related brain injury. Seizure burden correlates with adverse outcomes,^12–14^ and many electrographic seizures go undetected without monitoring.^15,16^ Beyond seizure detection, careful evaluation and classification of EEG background patterns offers valuable insight into neurologic function and can also help inform injury severity and prognosis.^16^ Near-infrared spectroscopy (NIRS) has also gained momentum as a non-invasive, bedside tool that estimates regional cerebral oxygenation and perfusion.^17^ Especially useful during transitional physiology, cardiac surgery, or periods of clinical instability, NIRS allows for early identification of cerebral desaturation or hemodynamic compromise. Although not diagnostic on its own,^18^ NIRS is frequently used alongside EEG in NNCC protocols, providing real-time information about cerebral oxygenation that complements EEG findings and helps guide clinical decision-making.^19^

### Neuroimaging

Cranial ultrasound is a first-line imaging modality for bedside detection of major structural abnormalities and for monitoring intracranial hemorrhage and ventricular dilation. However, MRI offers more detailed characterization of subtle or diffuse brain injury and is commonly used in high-risk populations, including infants with encephalopathy, seizures, congenital brain anomalies, congenital heart disease, and extreme prematurity. T1- and T2-weighted sequences, diffusion-weighted imaging (DWI), and susceptibility-weighted imaging (SWI) can detect evolving injury. In term infants, DWI abnormalities after perinatal hypoxic-ischemic injury typically peak around days 4–6 of life.^20^ Standardized MRI scoring systems help translate imaging findings into predictions about future development, supporting early decisions about treatment, follow-up, and family counseling.^21,22^

### Neurodevelopmental assessments

Early neurodevelopmental assessments are integrated into NNCC to relate imaging and neuromonitoring findings to a child’s functional development. Tools such as the General Movements Assessment (GMA) and structured neurological exams like the NICU Network Neurobehavioral Scale (NNNS) and Hammersmith Neonatal Neurological Exam (HNNE) can detect mild motor deficits or early signs of cerebral palsy.^23–25^ Feeding behavior and parent-infant interaction are also critical indicators of early brain function and correlate with neurological outcomes.^26,27^ Many NICUs are beginning this process before discharge, linking hospital-based care with long-term developmental follow-up.

### Neuro-focused interdisciplinary team

**Fig. 2.**
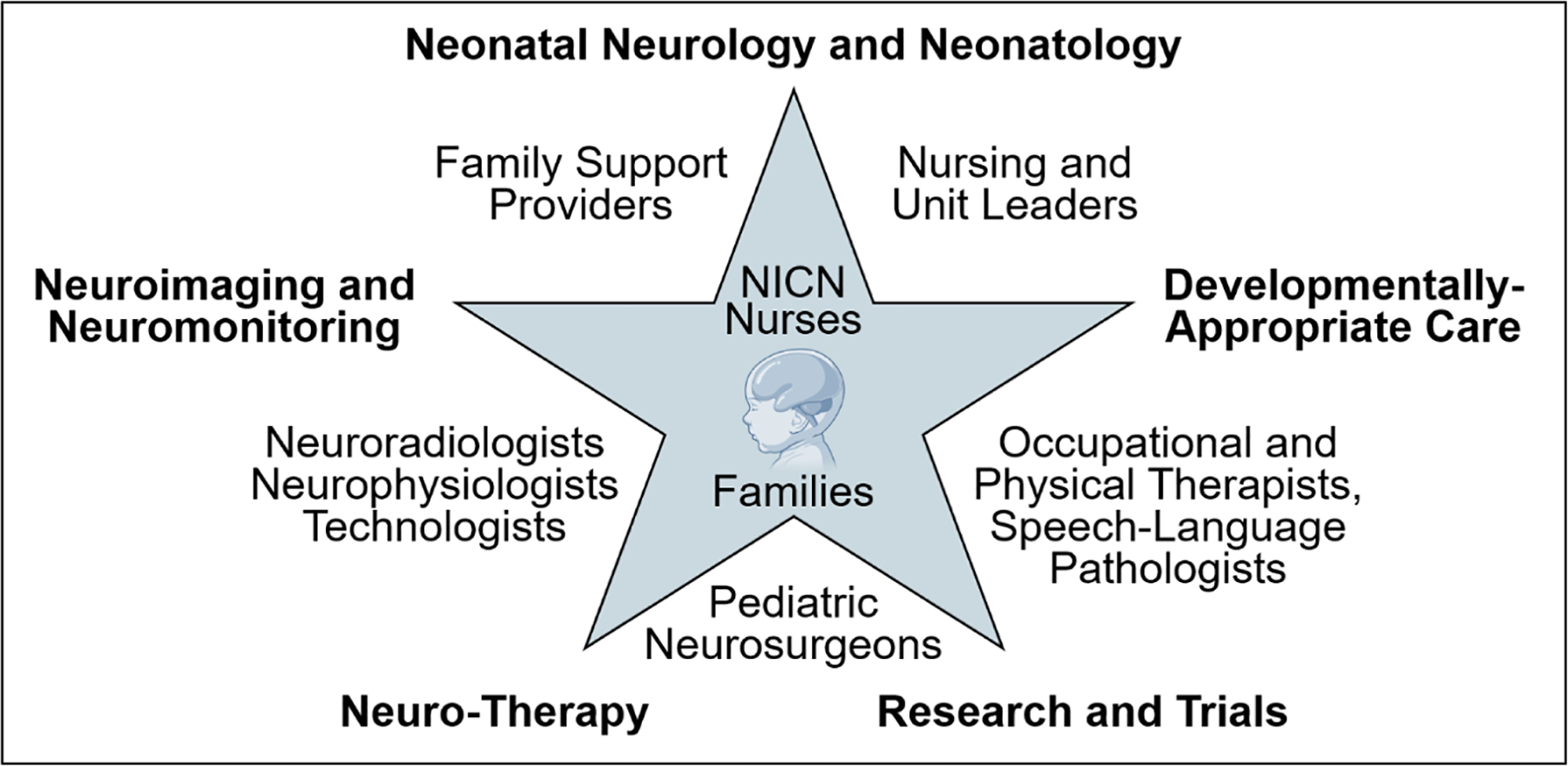
Multidisciplinary neuro-intensive care nursery (NICN) team components and care domains. Central image created in BioRender. Petersen, M. (2025) https://BioRender.com/eevknb8.

The NNCC model is built on a multidisciplinary team that includes neonatologists, neonatal/pediatric neurologists, neurophysiologists, neuroradiologists, neurosurgeons, NICN-trained nurses, developmental specialists, and family support providers such as social workers, care coordinators, psychologists, and parent liaisons (Fig. 2).^4^ In some centers, dedicated NICN teams conduct daily rounds with a “brain-first” framework. Elsewhere, shared protocols help ensure neurological risks are consistently identified and managed, even in settings without subspecialty teams. To support reliable and timely delivery of brain-focused care, many NICUs have embedded NNCC practices into structured quality improvement initiatives that use clinical care bundles or integrated neuroprotective and developmental care pathways.^28^ Although NNCC was initially emphasized in term infants with HIE, it now extends to a broader range of high-risk newborns, including preterm infants, cardiac and surgical patients, and those with genetic or metabolic encephalopathies.

## NNCC improves outcomes

One of the most significant advances in NNCC has been in the detection and management of seizures. cEEG monitoring has become standard practice in many NNCC settings, enabling the identification of electrographic seizures that would otherwise go unnoticed.^16^ Early and targeted seizure management has been shown to reduce overall seizure burden, which is strongly linked to adverse cognitive and motor outcomes later in life.^12,29^ The implementation of structured seizure response protocols has further improved care by reducing the time to antiseizure medication administration.^30^ These advances have also led to more precise treatment strategies, minimizing overtreatment and limiting unnecessary exposure to antiseizure medications,^31–33^ an important shift given the potential neurotoxicity of these agents on the developing brain.

In preterm infants, NNCC-aligned practices, such as tighter control of cerebral oxygenation, minimization of hemodynamic instability, and less invasive respiratory support, have been linked to reduced rates of IVH and improved white matter integrity.^34,35^ These strategies are often delivered through structured care bundles, such as IVH prevention protocols, which commonly include clinical care recommendations like cautious administration of intravenous fluids, midline head positioning, and reduced handling in the immediate postnatal period.^36–38^ By incorporating neuromonitoring and early neuroimaging into routine care, NNCC also helps detect brain injury in its earliest stages and supports timely interventions aimed at stabilizing brain function and minimizing further injury. These efforts are especially critical given the vulnerability of the preterm brain and the strong correlation between early brain injury and long-term neurodevelopmental disability.^39,40^

NNCC has also strengthened prognostic precision in neonatal care. The use of MRI during the neonatal period enables early detection of injury patterns associated with later outcomes. For example, deep gray matter injury has been correlated with cerebral palsy and motor delay at school age,^41^ allowing for earlier referral to developmental therapies and better-informed family counseling. Taken together, these improvements reflect the power of an organized, neurologically focused care model to reduce injury and improve developmental trajectories via individualized care and early intervention. As NNCC becomes more widely adopted and refined, its role in shaping neonatal outcomes continues to grow.

## Expanding the NNCC continuum

NNCC is no longer confined to the walls of the NICU. It has evolved into a comprehensive, lifespan-informed framework that begins before birth and extends through the early years of life, encompassing periods of both heightened vulnerability and extraordinary neuroplasticity.^34^ This continuum-based model marks a fundamental shift in neonatal care, emphasizing the importance of brain-focused support from prenatal diagnosis through long-term developmental follow-up. By connecting fetal assessments, neonatal interventions, and coordinated outpatient care, NNCC enables earlier recognition of neurological risk, more personalized care strategies, and a sustained focus on optimizing outcomes over time.

### Fetal brain care

The fetal period is a time of rapid neurodevelopment, during which disturbances in oxygenation, perfusion, inflammation, or molecular signaling pathways can have lasting effects on brain structure and function. Advances in fetal MRI and neurosonography have significantly improved the prenatal detection of cerebral anomalies, including ventriculomegaly, cortical malformations, callosal agenesis, cerebellar hypoplasia, intracranial hemorrhage, and white matter abnormalities.^42–44^ MRI diffusion imaging and volumetric analysis allow for detailed assessment of brain maturation and emerging connectivity,^45–47^ enhancing diagnostic precision and informing prenatal counseling, risk stratification, and delivery planning. These innovations are helping to usher in the field of fetal neurology, a rapidly evolving discipline aimed at understanding, monitoring, and ultimately optimizing brain development before birth.^48–50^ Fetal neurology consultations and structured care pathways are helping to connect prenatal diagnoses with postnatal NNCC management, ensuring early neurocritical needs are addressed and longer-term developmental planning is initiated without delay

In parallel, several antenatal therapies have been integrated into care for pregnancies at high neurological risk. Magnesium sulfate, administered to women at risk for preterm delivery, has consistently shown neuroprotective effects, reducing rates of IVH, cerebellar hemorrhage, and severe motor impairment.^51,52^ Corticosteroids, long used for promoting fetal lung maturation, have been associated with a reduced incidence of IVH in preterm infants when given before early delivery.^53^ Delayed cord clamping at birth has also been shown to lower the risk of IVH as well as improve neonatal iron stores and enhance early myelination,^54,55^ benefiting both immediate stability and long-term neurodevelopment. Melatonin is currently under investigation as another fetal neuroprotective agent, potentially acting through antioxidant pathways to support white matter development.^56^ Additionally, fetal surgery has shown promise in improving neurological outcomes in select populations. The Management of Myelomeningocele Study (MOMS) demonstrated that in utero repair of myelomeningocele leads to better motor function and reduced need for shunting compared to postnatal repair.^57^ Preclinical gene therapy studies and early in utero enzyme replacement trials for lysosomal storage diseases are also opening new avenues to prevent neurological injury at the molecular level before birth.^58,59^ These strategies reflect a broader shift in NNCC toward proactive interventions that prevent brain injury and promote normal neurodevelopment.

### Long-term follow-up

As NNCC evolves into a longitudinal model, long-term neurodevelopmental monitoring has become a critical priority, bridging hospital discharge with early childhood care. Many infants with perinatal brain injury or neurological risk require ongoing surveillance, as developmental challenges may emerge gradually or change over time.^60^ Dedicated neonatal neurodevelopmental programs offer structured, serial assessments that track growth and function across key domains.^2,4^ Standardized tools such as the Bayley Scales of Infant and Toddler Development and the Hammersmith Infant Neurological Examination are commonly used to evaluate cognitive, language, and motor development, while additional assessments may address executive function, social-emotional skills, and adaptive behavior.^61–63^ These programs enable early detection of evolving deficits and support individualized care aligned with family-centered goals, linking children and caregivers to early intervention services, behavioral health, parental support, and additional subspecialty services, such as developmental-behavioral pediatrics or physiatry, if needed. By extending brain-focused care into the post-discharge period, the partnership with longitudinal follow-up programs strengthens the continuum of NNCC and maximizes the opportunity for each child to reach their full developmental potential.

## Emerging technologies in NNCC

Emerging technologies in NNCC are rapidly advancing in research settings, offering glimpses of how neurocritical care might look in the future. While most applications remain investigational, these tools and techniques may enhance the ability to assess neurological risk, detect injury at earlier stages, and tailor care strategies to the unique physiologic and genetic profile of each infant. In particular, technological advances may have the greatest impact in extending neurocritical care practices to settings with limited subspecialty expertise. Taken together, these innovations point toward a future in which NNCC for at-risk newborns is more precise and more accessible.

### Refining ultrasound techniques

Bedside Doppler-based vascular flow studies are gaining interest as a noninvasive approach to assessing cerebral hemodynamics in critically ill infants.^64,65^ Low resistive indices on Doppler have been associated with more severe HIE and worse long-term neurodevelopmental outcomes.^66,67^ In preterm infants, pulsatile flow in the internal cerebral vein has been linked to increased risk of IVH,^68,69^ suggesting that Doppler-based measures may serve as early markers of brain injury risk. In parallel, the use of point-of-care ultrasound (POCUS) and targeted neonatal echocardiography to assess cardiac output and systemic perfusion has expanded in the NICU.^70^ These tools may allow clinicians to connect cerebral and systemic physiology in real time. Together, these advances can help transform ultrasound into a dynamic neuromonitoring modality, paving the way for more individualized neuro-critical care.

### Expanding MRI capabilities

MRI has advanced beyond structural imaging to encompass techniques that reveal critical aspects of brain development, connectivity, and metabolism. Diffusion tensor imaging (DTI) and tractography allow more detailed visualization of white matter architecture, offering early insights into disruptions in sensorimotor and cognitive networks.^71,72^ Additional research sequences, such as T1/T2 ratio mapping and MR fingerprinting-derived myelin water fraction, are being integrated into neonatal protocols to better quantify myelin content and detect early deviations linked to long-term outcomes.^73,74^ Resting-state functional MRI (fMRI) captures spontaneous fluctuations in cerebral blood oxygenation to map emerging brain networks,^75,76^ which may provide new imaging biomarkers of brain injury and neurodevelopmental risk. Magnetic resonance spectroscopy (MRS) enables metabolic profiling of brain tissue, with alterations in choline, lactate, and N-acetylaspartate offering insight into energy metabolism, injury severity, and prognosis.^77–79^ Together, these techniques may help characterize the functional impact of early brain injury and will be particularly valuable for assessing the effects of prematurity, hypoxia, and congenital anomalies on brain development.

### Advances in multimodal neuromonitoring

Traditional NIRS has provided valuable trends in regional brain oxygenation, but newer experimental approaches are expanding physiologic insight. Frequency-domain NIRS (FDNIRS) improves upon conventional NIRS by enabling absolute quantification of oxy- and deoxyhemoglobin concentrations, improving measurement reliability.^80,81^ When integrated with diffuse correlation spectroscopy (DCS), an optical technique that measures cerebral blood flow by detecting light scattering from moving red blood cells, FDNIRS/DCS provides a more complete picture of cerebral oxygen delivery and consumption.^80,81^ These technologies can be integrated with EEG, heart rate variability, and other continuous data streams to create a multimodal approach to brain monitoring. This integration could support earlier recognition of impaired autoregulation and hemodynamic compromise, potentially allowing for real-time adjustment of interventions during critical care, therapeutic hypothermia, or perioperative periods.

### Artificial intelligence (AI) and machine learning (ML)

AI and ML are emerging tools in NNCC research, with growing potential to support interpretation of complex physiological and neurological data in the NICU. AI-based EEG platforms have been explored for continuous, automated seizure detection, offering near expert-level neurophysiologic interpretation in some experimental and pilot settings.^82,83^ These systems may help reduce time to diagnosis and treatment, particularly in units without on-site neurology support. ML approaches are also under investigation for predicting seizure onset in infants with conditions such as HIE,^84^ opening the door to more proactive monitoring and earlier therapeutic intervention. Additionally, AI tools have shown promise in automated classification of EEG background activity, a task that traditionally requires specialized expertise. For example, platforms like BabaCloud generate a quantitative Brain State of the Newborn (BSN) score, translating complex EEG background patterns into an interpretable scale that reflects encephalopathy severity and maturational trends.^85–87^ This could support longitudinal tracking of brain function and decision-making in centers with limited neurophysiology resources. In parallel, ML algorithms trained on vital sign patterns—such as heart rate variability metrics like the HeRO score—are being studied for their ability to detect early physiologic instability before clinical deterioration and to potentially link physiologic trends with long-term neurodevelopmental risk.^88^ While still primarily in the research domain, these AI/ML-based technologies hold promise for more personalized neuroprotective care, especially in resource-limited or high-acuity settings.

### The retina as a window to the developing brain

The retina serves as an accessible extension of the central nervous system, offering a non-invasive window into neurodevelopment. Optical coherence tomography (OCT), adapted for use in preterm infants, enables high-resolution imaging of retinal layers and vasculature, offering insight into neural and vascular maturation during critical developmental windows.^89,90^ Retinal abnormalities in preterm infants, such as reduced nerve fiber layer thickness, have been associated with later impairments in neurodevelopmental outcomes.^89^ While still investigational, OCT may eventually complement traditional ROP examinations by offering additional insight into early structural changes and potential markers of neurodevelopmental vulnerability. Accordingly, detailed retinal imaging holds promise as a scalable method for early monitoring of neurodevelopmental risk in the NICU.

### Genomic technologies guiding precision NNCC

Rapid advances in sequencing technology have made comprehensive genomic analysis feasible in critically ill neonates. Whole-exome and whole-genome sequencing can now be completed within days, enabling earlier diagnosis of neurological disorders, including genetic epilepsies, metabolic encephalopathies, and syndromic malformations.^91–94^ Genomic insights are increasingly used to tailor therapeutic strategies, such as more precise use of antiseizure medications, and better inform long-term prognosis. In cerebral palsy—traditionally viewed as an acquired condition—genomic sequencing identifies an underlying genetic contributor in roughly 30 % of cases, with 8 % prompting changes in clinical care.^95,96^ Integrating genetic data with neuroimaging and clinical findings further strengthens the ability to guide care from the earliest stages and lays the foundation for a more personalized model of NNCC. Moreover, the emergence of viral- and CRISPR-based “genomic surgery” and gene therapies opens the possibility of corrective treatments for genetic neurologic disorders previously regarded as incurable,^97^ setting the stage for a new era in disease-modifying interventions in NNCC.

## Neurotherapeutic horizons in NNCC

### Neuroprotective agents for HIE

While therapeutic hypothermia remains the cornerstone of treatment for moderate-to-severe HIE, it offers incomplete protection, particularly in infants with delayed access to care or comorbidities.^20^ Adjunctive therapies have been explored to extend neuroprotection, but results have been mixed. Erythropoietin (EPO), which showed initial promise in preclinical studies and early-phase trials, did not improve neurodevelopmental outcomes in the large randomized controlled *HEAL* trial.^98^ Similarly, inhaled xenon did not improve MRI biomarkers of brain damage or lessen mortality.^99^ These findings have prompted a shift toward alternative strategies that address oxidative stress, mitochondrial dysfunction, and neuroinflammation. Potential neuroprotective agents for HIE such as melatonin, allopurinol, and caffeine are currently under investigation in clinical trials.^100,101^ Optimizing therapeutic timing and combining adjunctive agents with complementary mechanisms may be necessary to maximize clinical benefit in conjunction with therapeutic hypothermia.

### Blood-targeted strategies for IVH

IVH remains a major source of morbidity in preterm infants, due both to the primary hemorrhagic insult and to the secondary effects of toxic blood components on periventricular development.^102^ The blood clotting protein fibrinogen has been shown to disrupt key developmental signaling pathways in animal models of preterm brain injury—specifically by altering the balance of sonic hedgehog (SHH) and bone morphogenetic protein (BMP) signaling in the cerebellum and forebrain—leading to impaired neurogenesis and myelination.^103–105^ Concurrently, the breakdown of hemoglobin releases free iron into the ventricular system and choroid plexus, driving oxidative stress, inflammation, and white matter injury.^106,107^ These mechanisms may contribute to post-hemorrhagic ventricular dilatation and broader neurodevelopmental impairment.^102^ Targeted interventions under pre-clinical investigation include fibrinogen pathway modulators, iron chelators, and agents that regulate choroid plexus ion transport to reduce cerebrospinal fluid accumulation.^106,108,109^ Early blood clear ance has also become a therapeutic focus. The *DRIFT* (Drainage, Irrigation, and Fibrinolytic Therapy) trial showed that active removal of intraventricular blood resulted in significantly improved cognitive outcomes at school age.^110^ However, the technical complexity and risk of rebleeding limited widespread adoption. Building on this, the *ENLIVEN-UK* trial is evaluating neuro-endoscopic lavage as a less invasive method of early clot removal, aiming to reduce the need for permanent CSF diversion and improve neurodevelopmental outcomes.^111^

### Myelin-supportive therapies in preterm infants

Diffuse WMI is a major contributor to neurodevelopmental impairment in preterm infants, which largely stems from arrested oligodendrocyte progenitor cell (OPC) maturation and disrupted myelination.^112^ Among existing therapies, caffeine has demonstrated neuroprotective properties beyond respiratory stimulation, with findings from the *CAP* trial and subsequent imaging studies suggesting improved white matter development and long-term cognitive outcomes.^113,114^ EPO, which has been shown to promote OPC survival and maturation,^115^ did not improve myelination or neurodevelopmental outcomes in large clinical trials of preterm infants.^116–118^ More recently, clemastine, an over-the-counter antihistamine with anticholinergic properties, has emerged as a promising promyelinating agent due to its ability to accelerate OPC differentiation and remyelination in white matter disease models.^119^ Additional compounds under investigation include thyroid hormone, the cholesterol-like compound olesoxime, and metformin, all of which have shown efficacy in promoting myelination in preclinical models.^120^ Together, these therapies reflect a growing shift toward targeted support of white matter maturation to improve neurological outcomes in preterm infants.

### Stem cell–based therapies

Stem cell approaches represent a compelling avenue for both neuroprotection and repair across neonatal brain injury. In particular, mesenchymal stem cells (MSCs), often derived from umbilical cord or placental tissue, have been evaluated for their immunomodulatory and trophic effects in models of HIE, IVH, and neonatal stroke and in early clinical trials.^121–123^ Rather than relying on long-term engraftment, these therapies are thought to act largely through paracrine signaling via the release of extracellular vesicles (EVs) which carry anti-inflammatory, angiogenic, and neurotrophic factors.^123^ Intranasal delivery has emerged as a non-invasive method to deliver MSCs/EVs directly to the CNS, and early-phase human trials are underway to assess safety and feasibility in perinatal arterial ischemic stroke.^122^ Critical challenges remain in standardizing cell source, dosing, and timing of administration, but this platform offers substantial promise as a cell-based intervention for evolving brain injury.

### Biomarkers of neonatal brain injury

Efforts to individualize care and optimize the timing of intervention in NNCC have fueled growing interest in identifying reliable biomarkers of neonatal brain injury. In HIE, serum inflammatory markers such as interleukin-6 (IL-6), IL-8, tumor necrosis factor-alpha (TNFα), interleukin-1-beta (IL-1β), and IL-1 receptor antagonist (IL-1RA) have been associated with encephalopathy severity, seizure burden, and abnormal MRI findings.^124–126^ Among emerging candidates, inter-alpha inhibitor proteins (IAIPs)—liver-derived protease inhibitors involved in immune modulation and vascular integrity—have shown particular promise. Reduced levels of IAIPs at birth have been linked to greater encephalopathy severity and adverse outcomes in cooled infants, suggesting a potential role for IAIPs as both a biomarker and a therapeutic target in HIE.^127^ Other candidate biomarkers include neuron-specific enolase (NSE), GFAP or S100B, and neurofilament light chain (NfL), which reflect neuronal, glial, and axonal injury, respectively.^126,128^ In preterm infants with IVH, elevated ferritin and hemoglobin breakdown products in the CSF may provide additional insight into secondary injury mechanisms.^129^ While no single biomarker has yet demonstrated sufficient specificity for routine use, combining molecular profiles with neuromonitoring and imaging could enhance early risk stratification and therapeutic precision. Large, multicenter validation studies remain essential for clinical translation.

## Challenges in NNCC

### Global barriers to implementation and effectiveness

Much of the foundational progress in NNCC has taken place in high-resource settings, where therapeutic hypothermia, advanced neuromonitoring, imaging, and multidisciplinary neurodevelopmental teams are readily accessible. In contrast, low- and middle-income countries (LMICs), which face a high burden of neonatal encephalopathy and preterm birth, often lack the infrastructure and clinical workforce to support comprehensive NNCC.^130^ The HELIX trial, a landmark multicenter study conducted in South Asia, found no neuroprotective benefit from therapeutic hypothermia and observed higher mortality among cooled infants, raising important questions about the applicability of interventions developed in different clinical contexts.^131^ Differences in infection burden, timing of care, and perinatal management may influence both the underlying pathophysiology and the response to neuroprotective therapies. These findings underscore the need for context-specific validation and adaptation of NNCC strategies, including the development of lower-cost tools to bring brain-focused care to settings with limited resources.

### Population-specific limitations

Even within high-resource settings, existing neuroprotective strategies are not universally effective across all neonatal populations. For example, the evidence base for therapeutic hypothermia, until recently, largely excluded preterm infants, yet these infants can present with encephalopathy and are vulnerable to similar patterns of injury. Emerging data suggest that cooling may offer less benefit—or even potential harm—in this group.^132^ Similarly, neonates with complex congenital anomalies, inborn errors of metabolism, or rare syndromes often fall outside the scope of standardized NNCC pathways. In these cases, generalized protocols may overlook the need for tailored diagnostic and therapeutic approaches based on underlying disease mechanisms. Improving outcomes for these high-risk populations will depend on prospective trials and focused observational studies that help match therapies to the specific biological processes driving neurologic disease.

### Transitions from acute to chronic care

While NNCC has become increasingly sophisticated in the acute hospital setting, continuity of care beyond discharge remains a major challenge. Many infants with neurological risk experience delays in accessing follow-up services, inconsistencies in developmental surveillance, or lack of coordination among care teams. These gaps can delay diagnosis of emerging neurodevelopmental concerns and reduce the effectiveness of early intervention. The transition from NICU to home is a critical juncture that requires reliable handoffs and clearly defined follow-up pathways. However, these processes are highly variable between centers and are particularly vulnerable in resource-limited or rural settings where specialty care is scarce. Strengthening long-term NNCC requires investment in integrated, family-centered systems that extend beyond the NICU and into early childhood.

### Communication and family engagement

Families play a central role in NNCC, yet communication with parents about neurological conditions remains a complex and emotionally charged process. Studies have shown that families often experience uncertainty or distress when navigating discussions about brain injury and the potential for future disability.^133,134^ Effective engagement requires accurate, transparent information as well as communication strategies that are responsive to the cultural contexts and emotional needs of the families. The ALIGN framework offers one model for structured family-centered communication in neurocritical care, emphasizing shared understanding, mutual trust, and alignment of goals.^135^ However, widespread implementation of such frameworks remains limited. Building communication competency and integrating it into medical training and interdisciplinary care is essential for improving parent experience and long-term trust in the healthcare system.

## Conclusion

NNCC represents more than a new set of tools or protocols—it reflects a fundamental shift in how we support brain health during the most critical stages of development. For centers seeking to establish an NNCC program, key initial steps are outlined in Box 2. By placing the brain at the center of neonatal care, NNCC promotes earlier recognition of neurological risk, more precise and physiology-guided intervention, and sustained focus on outcomes that extend well beyond the NICU. While advances in neuromonitoring, neuroimaging, genomics, and neurotherapeutics are driving the field forward, the future impact of NNCC will depend on how well we coordinate across disciplines, engage families in meaningful ways, and build systems that deliver consistent, high-quality care to all patients no matter where they live. As NNCC continues to grow, so too must our shared commitment to the belief that investing in the newborn brain is one of the most powerful ways we can shape a child’s future.

## Data Availability

No data was used for the research described in the article.

## Box 1. Why were Neuro-Intensive Care Nurseries (NICNs) / Neuro-NICUs developed?

1. **To Centralize and Standardize Neurologic Care**
  In a traditional NICU, neurologic issues are typically addressed reactively, inconsistently, or via consultation. NNCC created a dedicated infrastructure and team, including neonatologists, neurologists, nurses, and radiologists, focused on brain-directed care, including structured rounds and standardized protocols and guidelines.
2. **To Educate and Empower the Bedside Team**
  Nurses and trainees were given specialized training to recognize subtle neurologic changes, interpret neuromonitoring data, and collaborate more actively in neurologic management.
3. **To Support the Use of Therapeutic Hypothermia**
  As cooling therapy became standard for HIE, there was a growing need for a specialized care model that could monitor brain function during hypothermia and rewarming, using multimodal neuromonitoring and careful neurologic assessment.
4. **To Improve Detection and Treatment of Seizures**
  Many neonatal seizures are electrographic only, meaning they lack clinical signs and go undetected without EEG. NNCC units were designed to incorporate continuous EEG monitoring and neurologist input to identify and manage seizures in real time, especially in neonates with HIE, stroke, or metabolic disease.
5. **To Integrate Neuroimaging and Prognostication**
  NNCC units aimed to provide timely cranial ultrasound, MRI, and functional assessments that could support prognosis, guide care decisions and early interventions, and enhance communication with families, especially in high-risk term and preterm infants.
6. **To Align Acute Care with Long-Term Neurodevelopment**
  Early NNCC pioneers recognized that acute brain care influences lifelong outcomes, so they structured care to facilitate smoother handoffs to follow-up services, neurodevelopmental clinics, and early intervention programs.

## Box 2. Starting a neonatal neurocritical care (NNCC) program

1. **Gather key partners to develop a leadership structure and regular meeting schedule**
  - Neonatology
  - Child Neurology/Neonatal Neurology
  - Nursing
  - Parent partner
2. **Identify local resources**
  - Pediatric Neurosurgery
  - Pediatric Neurophysiology and Neuromonitoring availability (EEG, aEEG)
  - Pediatric Neuroradiology and MRI sequences tailored for the newborn brain
  - Bedside head ultrasound
  - Fetal consultations
  - Infant follow-up program
  - Genetic testing
3. **Craft guidelines accounting for availability of local resources. Examples include:**
  - Therapeutic hypothermia
  - Seizure management
  - Intraventricular hemorrhage and post-hemorrhagic hydrocephalus
  - Stroke
4. **Develop targeted education programs**
  - Residents (Child Neurology and Pediatric)
  - Fellows (Neonatology)
  - Nurses
  - Faculty
